# Voluntary exercise attenuates LPS-induced reductions in neurogenesis and increases microglia expression of a proneurogenic phenotype in aged mice

**DOI:** 10.1186/s12974-015-0362-0

**Published:** 2015-07-30

**Authors:** Alyssa M. Littlefield, Sharay E. Setti, Carolina Priester, Rachel A. Kohman

**Affiliations:** Department of Psychology, University of North Carolina Wilmington, 601 S. College Road, Wilmington, NC 28403-5612 USA; Department of Biology, University of North Carolina Wilmington, 601 S. College Road, Wilmington, NC 28403-5612 USA

**Keywords:** BDNF, Wheel running, Lipopolysaccharide, Spatial learning, Hippocampus

## Abstract

**Background:**

Microglia can acquire various phenotypes of activation that mediate their inflammatory and neuroprotective effects. Aging causes microglia to become partially activated towards an inflammatory phenotype. As a result, aged animals display a prolonged neuroinflammatory response following an immune challenge. Currently unknown is whether this persistent neuroinflammation leads to greater reductions in hippocampal neurogenesis. Exercise has been shown to alter microglia activation in aged animals, but the nature of these changes has yet to be fully elucidated. The present study assessed whether aged mice show enhanced reductions in hippocampal neurogenesis following an acute immune challenge with lipopolysaccharide (LPS). Further, we assessed whether voluntary wheel running protects against the effects of LPS.

**Methods:**

Adult (4 months) and aged (22 months) male C57BL6/J mice were individually housed with or without a running wheel for a total of 9 weeks. After 5 weeks, mice received a single intraperitoneal LPS or saline injection in combination with four daily injections of bromodeoxyuridine (BrdU) to label dividing cells. Tissue was collected 4 weeks later and immunohistochemistry was conducted to measure new cell survival, new neuron numbers, and microglia activation.

**Results:**

Data show that LPS reduced the number of new neurons in aged, but not adult, mice. These LPS-induced reductions in neurogenesis in the aged mice were prevented by wheel running. Further, exercise increased the proportion of microglia co-labeled with brain-derived neurotrophic factor (BDNF) in the aged.

**Conclusions:**

Collectively, findings indicate that voluntary wheel running may promote a neuroprotective microglia phenotype and protect against inflammation-induced reductions in hippocampal neurogenesis in the aged brain.

## Introduction

The adult brain retains the ability to generate new neurons in the hippocampus. While the precise function of these new cells has yet to be fully elucidated, evidence indicates they have relevance to cognitive function [1, 2]. New neurons display a unique hyper-excitability making it easier to induce long-term potentiation in new neurons, which likely improves their chances of survival and integration into existing neural circuits [3, 4]. Eventually, new neurons become mature granular cells that likely engage in all functional activities mediated by the granular cell layer.

Microglia are a dynamic group of immune cells that can modulate hippocampal neurogenesis [5–7]. However, whether microglia have beneficial or detrimental effects depends on whether the cells are expressing an inflammatory or neuroprotective phenotype. Microglia expressing the classic inflammatory phenotype can decrease neurogenesis [8]. Classically activated microglia release a host of inflammatory molecules including the proinflammatory cytokines interleukin-1β (IL-1β) and tumor necrosis factor-α, both of which have been shown to reduce hippocampal neurogenesis [9, 10]. In contrast, microglia expressing the resting or alternative neuroprotective phenotype can stimulate stem cell proliferation, direct migration, and increase neurogenesis [11, 12, 7]. These supportive effects of alternatively activated microglia may relate to the release of anti-inflammatory cytokines, such as interleukin-4 and interleukin-10 as well as neurotrophic factors including brain-derived neurotrophic factor (BDNF) and insulin-like growth factor (IGF-1) [13–16].

Normal aging primes microglia towards the classic inflammatory phenotype, which increases basal release of the proinflammatory cytokines IL-1β and interleukin-6 [17]. In addition, in conditions of normal and pathological aging microglia show an increase in proliferation, a response associated with the classic inflammatory phenotype [18, 15]. This age-related increase in neuroinflammation partially contributes to basal reductions in neurogenesis, as inhibition of IL-1β increases neurogenesis in aged subjects [19]. However, whether aged subjects show a greater reduction in hippocampal neurogenesis following an immune challenge is unknown.

One intervention that enhances neural plasticity as well as modulates immune activity is exercise [15, 20, 1]. A wealth of evidence indicates that exercise enhances hippocampal neurogenesis in both adult and aged subjects [15, 1, 21]. The beneficial effects of exercise likely relate to changes in a host of different signaling systems, including increased production of neurotrophic factors such as BDNF and IGF-1, altered levels of endogenous opioids, decreased levels of bone morphogenetic protein, and potentially decreased inflammation [22–26]. Prior work has shown that exercise-induced enhancements in hippocampal neurogenesis and spatial learning in middle-aged mice were accompanied by elevated hippocampal levels of BDNF [24]. In regards to immune function, wheel running has been shown to reduce expression of markers associated with the classic inflammatory microglia phenotype in the aged brain, as running decreased basal expression of IL-1β and tumor necrosis factor-α in aged rats [27–29]. In addition, wheel running attenuates microglia proliferation in aged mice [15]. In adults, prior reports have shown that exercise increases microglia proliferation in select cortical regions and the dentate gyrus [30, 31]. However, exercise had no effect on microglia activation in the hippocampus of adult mice, as measured by cell morphology and assessment of several classic microglia phenotype markers [31]. Both adult and aged mice show an increase in the proportion of IGF-1 positive microglia following wheel running, potentially indicating that exercise induces a phenotype that may support new cell survival [15]. Taken together these data indicate that exercise can alter microglia activation, but the effects may depend on the organism’s age. Whether exercise-induced changes in microglia activation contribute to the increases in hippocampal neurogenesis is presently unknown.

The objective of the present study was to determine whether aged mice show greater reductions in hippocampal neurogenesis following an immune challenge with the bacterial endotoxin, lipopolysaccharide (LPS). In addition, we assessed whether exercise attenuates LPS-induced deficits in hippocampal neurogenesis. Finally, we assessed whether exercise would alter the proportion of microglia that express BDNF.

## Methods

### Experimental subjects

Subjects were 21 adult (4-month-old) and 21 aged (22-month-old) male C57BL6/J mice purchased from the National Institute on Aging rodent colony maintained by Charles River. Mice were individually housed, given *ad libitum* access to food and water, and housed under a reverse 12 h light/dark cycle. Animals were treated in compliance with the *Guide for the Care and Use of Laboratory Animals* and the experiment was conducted in accordance with a protocol approved by the Institutional Animal Care and Use Committee (IACUC) at the University of North Carolina Wilmington.

### Experimental design

**Fig. 1:**
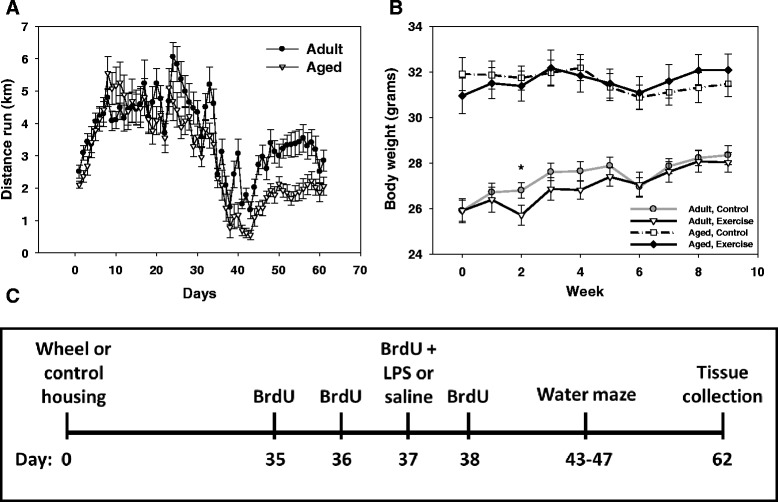
Distance run, body weight, and experimental timeline. Average distance (km) run per day by adult and aged mice over nine weeks of running wheel access (**a**). Average body weight across the nine weeks of the experiment (**b**). * indicates a significant difference in body weight between adult control and adult exercise mice. Lines represent group means ± standard error of the mean (SEM). The experimental timeline details the timing of wheel access, BrdU and LPS or saline injections, behavioral testing, and tissue collection (**c**)

Within an age group mice were divided into either the exercise (access to a running wheel) or control condition. Mice in the control condition were individually housed in polypropylene cages (30 cm L × 15 cm W × 20 cm H). Mice in the exercise condition were individually housed in polypropylene cages (36 cm L × 20 cm W × 14 cm H) with a 23 cm diameter running wheel (Respironics, Bend, OR). Wheel rotations were continuously collected in 1 min intervals via magnetic switches interfaced to a computer using Vital View software (Respironics, Bend, OR). To maintain normal levels of activity in the control mice, we opted not to include a locked wheel condition, as mice increase their physical activity levels by climbing on the wheels [32, 33]. Mice were housed under control or exercise housing conditions for a total of nine weeks (see Fig. 1c for experimental timeline). All mice were weighed once a week. Five weeks into the exercise or control housing condition, all mice received four consecutive intraperitoneal injections of bromodeoxyuridine (BrdU; 75 mg/kg), a thymidine analogue that incorporates into dividing cells. In addition, the mice received an intraperitoneal injection of lipopolysaccharide (LPS; 250 μg/kg) or saline. The LPS or saline injection was given on the third day of BrdU administration, so all animals received BrdU injections two days prior to, on the day of, and one day after LPS or saline administration for a total of four BrdU injections (see Fig. 1c). There was a total of eight treatment groups: adult control saline, adult control LPS, adult exercise saline, adult exercise LPS, aged control saline, aged control LPS, aged exercise saline, and aged exercise LPS. Four weeks after the LPS or saline injection tissue samples were collected.

### Water maze testing

Six weeks into the exercise or control housing, mice were tested in the water maze to assess spatial learning. The maze consisted of a circular tub (121 cm diameter) and a white circular platform (12.7 cm). The platform was submerged 1 cm under the surface of the water. The water was made opaque with white tempera paint to conceal the platform. Water temperature was maintained at 19° ± 1 °C throughout testing. Extra-maze cues were located around the maze. Mice received three trials (up to 60 s) per day from one of four different start locations for five consecutive days. If a mouse failed to locate the platform within the allotted 60 s they were gently guided to the platform. All mice remained on the platform for 10 s at the end of each trial. A video tracking system (Topscan, Clever Systems, Reston, VA) was used to measure distance swam (mm), latency to locate the platform (seconds), and swim speed (mm/s). A single 60 s probe trial was conducted approximately two hours after the subject’s last trial on day 5. The platform was removed and the number of times the animal crossed over the original platform location as well as percent time spent in the four quadrants of the water maze (i.e., target, opposite, left, and right) were recorded by the tracking system.

### Immunohistochemistry and image analysis

#### Perfusions

Following the 9 weeks of wheel running or control housing, all mice were transcardially perfused with 4 % paraformaldehyde. Brains were fixed overnight in 4 % paraformaldehyde and then transferred into 30 % sucrose solution. Brains were sectioned at 40 μm on a cryostat.

#### New cell survival

All sections from a one-in-six series were stained for BrdU to identify new cells. Sections were spaced 240 μm apart and included sections from the complete rostral-caudal extension of the dentate gyrus. Briefly, free floating sections were rinsed in tissue buffering solution (TBS) and then treated with 0.6 % hydrogen peroxide for 30 min. To denature DNA, sections were placed in a solution of 50 % de-ionized formamide for 120 min at 65 °C, followed by 10 % 20× SCC buffer for 15 min, then 2 N hydrochloric acid for 30 min at 37 °C, and then 0.1 M boric acid (pH 8.5) for 10 min. Sections were blocked with a solution of 0.3 % Triton-X and 3 % goat serum in TBS (TBS-X plus) for 30 min, and then incubated with the primary antibody rat anti-BrdU (1:200; AbD Serotec, Raleigh, NC,USA) in TBS-X plus for 72 h at 4 °C. After washing with TBS, sections were blocked with TBS-X plus for 30 min and then incubated with a biotinylated goat anti-rat secondary antibody (1:250) in TBS-X plus for 100 min at room temperature. Sections were then treated with the ABC system (Vector, Burlingame, CA, USA) and stained using diaminobenzidine as the chromogen (Sigma, St. Louis, MO, USA). To obtain estimates of the number of new cells (BrdU+), the entire granular layer (bilateral), from all sections in the one-in-six series, was imaged by systematically advancing the field of view of a Zeiss brightfield light microscope by an individual blinded to the experimental conditions. Multiple non-overlapping photographs were taken to capture the entire granular cell layer, including dorsal and ventral hippocampal sections for all animals, via Axiocam interfaced to a computer, under 20× magnification. Bilateral images of the granular cell layer were captured from each section. Images were analyzed by ImageJ software [2, 21]. For each image, the granular layer was traced using the ImageJ software, and BrdU-positive nuclei were automatically counted by setting a fixed threshold to remove the background. The threshold selected was validated by comparing automated counts to hand counts. In addition, the area (pixels) of the granular cell layer within the trace was measured. Volume of the granular cell layer was determined by summing the traced area (pixels) from all sections for an individual sample. The summed areas were then converted from pixels to micrometers and multiplied by the section thickness (40 μm). Estimates of the total number of BrdU positive cells are expressed per cubic micrometer dentate gyrus sampled. Methodologies used to determine volume and quantify the number of BrdU positive cell number are based on prior reports [34, 2, 21, 15]. Values were further adjusted by removing the fraction of cells predicted to cross the boundary of the section on one side to produce unbiased estimates.

#### Immunofluorescence: Hippocampal neurogenesis

To determine the differentiation of the new cells, sections from one-in-six series were triple-labeled with rat anti-BrdU (1:200; Abcam Inc., Boston MA), the mature neuron marker mouse anti-neuronal nuclei (1:1000; Abcam Inc.), and the astrocyte marker rabbit anti-S100β (1:200; Abcam Inc.). Free-floating sections were handled as described above (New cell survival section) with the exception of the use of a cocktail of primary antibodies. Fluorescent markers (Alexafluor405, Alexafluor488, and Alexafluor647) were conjugated to secondary antibodies, made in goat, at a dilution of 1:200 and were also delivered as a cocktail. The hydrogen peroxide, ABC, and chromogen steps were omitted. An Olympus FV 1000 laser scanning confocal microscope (using a 40X oil objective) was used to determine the average group proportion of BrdU positive cells that differentiated into neurons as indicated by co-expression of neuronal nuclei or astrocytes as indicated by co-expression of S100β in the granular cell layer of the dentate gyrus. Images were taken by an individual blinded to the experimental conditions and included dorsal and ventral hippocampal sections for all groups. The group proportions were based on a minimum of 50 BrdU+ cells per group [35]. The group proportions were then multiplied by the number of BrdU positive cells to estimate the number of BrdU positive cells that became new neurons or astrocytes [35, 30, 1, 36]. The number of cells that co-labeled with BrdU and the astrocyte marker S100β within the granular cell layer was extremely limited and did not reach sufficient numbers to get an accurate estimate of the proportion of new cells that differentiated into astrocytes and therefore the data were not analyzed. While numerous S100β and BrdU co-labeled cells were observed in the surrounding hippocampus structures they were outside of the granular cell layer and were therefore not counted. Data presented show the number of new neurons (NeuN+ and BrdU+ cells).

#### Immunofluorescence: Proportion of microglia positive for BDNF

A one-in-six-series was double-labeled with the microglia/macrophage marker rabbit anti-Iba-1 (ionized calcium-binding adapter molecule 1; 1:500; Wako chemicals, Richmond, VA) and goat anti-BDNF (1:100; Santa Cruz Biotechnology, Santa Cruz, CA). Free-floating sections were handled as described above (Hippocampal neurogenesis section) with the exception of the use of donkey serum and fluorescent tagged (Alexa Fluor 405 and Alexa Fluor 647) secondary antibodies made in a donkey. An Olympus FV 1000 laser scanning confocal microscope (using a 40X oil objective) was used to identify the proportion of microglia (Iba-1+) that co-labeled with the neurotrophic factor BDNF in the dentate gyrus proper. Images were taken by an individual blinded to the experimental conditions. On average, 265 Iba-1 positive cells were analyzed within the five dorsal hippocampal section that were imaged (approximately −1.1 to −2.7 relative to bregma) per animal. Within each section, the dentate gyrus (including the granular cell layer, subgranular zone, polymorphic layer, and molecular layer) were imaged. When necessary, localization of the dentate gyrus was completed using a transilluminator lamp setting on the confocal microscope. Inclusion of a cell as an Iba-1 positive cell required that the cell body was labeled and fully visible. All Iba-1 positive cell bodies were included in the count. The percentage of Iba-1+ cells that co-labeled with BDNF was calculated by dividing the number of cells that co-labeled with Iba-1 and BDNF by the number of Iba-1 positive cells analyzed and multiplied by 100. It is important to note that the current study used Iba-1 which labels both microglia and macrophages. Therefore, it is possible that a portion of the cells we identified were macrophages. However, the impact of this is likely limited, as both cells mount similar immune responses and prior work has shown that microglia account for over 97 % of the CD11b positive cells isolated from the aged brain [37].

### Statistical analysis

Body weight data were analyzed by repeated measures ANOVA with age (adult or aged) and exercise condition (exercise or control) as the between-subject factors and week as the within-subject factor. Wheel running data were analyzed by repeated measures ANOVA with age as the between-subject factor and day as the within-subject factor. Changes in wheel running distance following LPS or saline were analyzed by repeated measures ANOVA with age and treatment (LPS or saline) as the between-subject factors and hour post treatment as the within-subject factor. Water maze data were analyzed by repeated measures ANOVA with age, exercise condition, and treatment as the between-subject factors and test day (days 1–5) as the within-subject factor. Immunohistochemistry data, change in body weight following treatment, and probe trial water maze data were analyzed by 2×2×2 ANOVAs with age, exercise condition, and treatment as the between-subject factors. Data for the number of BrdU positive cells and number of BrdU and neuronal nuclei co-labeled cells were not normally distributed and were therefore log transformed to correct normality as tested by Shapiro-Wilks test. Tukey’s test was used as the post hoc test to determine significant differences between groups. Correlations between new neurons with BDNF and Iba-1 co-labeled cells were conducted using Pearson’s r. For the correlations, only new neurons from the dorsal hippocampus (approximately −1.1 to −2.7 relative to bregma) were included to make the sampling regions comparable to the BDNF+ Iba-1 cells. An alpha level of p < 0.05 was considered statistically significant.

## Results

### Body weight

A significant main effect of age (F(1, 38) = 71.98, *p <* 0.0001) showed that aged mice weighed more than the adults (see Fig. 1b). A significant age × exercise condition × week interaction (F(1, 342) = 2.32, *p <* 0.05) showed that all mice gained weight during the nine weeks, but that the adult exercise mice weighed less than the adult controls during the second week of the experiment. A significant main effect of treatment (F(1, 34) = 75.56, *p <* 0.0001) showed that LPS-treated mice lost approximately 1.5 g of their starting body weight twenty-four hours after the injection. The weight loss induced by LPS administration was transient, as no long-term weight loss was observed in the LPS-treated mice when compared to the saline controls. Neither age nor exercise altered the response to LPS.

### Wheel running data

A significant age × day interaction showed that on select days during the 9 weeks of exercise the adult mice ran a longer distance than the aged mice (F(1, 1080) = 3.21, *p <* 0.05, see Fig. 1a). Overall the adult mice ran an average of 3.61 km/day and the aged mice ran 2.99 km/day. The decrease in distance ran around day 35 corresponds to the beginning of injections followed by behavioral testing a week later. Analysis of distance ran 0, 24, 48, and 72 h following an LPS or saline injection showed that LPS administration significantly decreased distance ran in both adult and aged mice (F(3, 48) = 5.51, *p <* 0.005, data not shown). There were no long lasting effects of LPS on wheel running, as seventy-two hours following LPS administration distance ran in both adult and aged mice no longer differed from saline-treated mice.

### Water maze

**Fig. 2:**
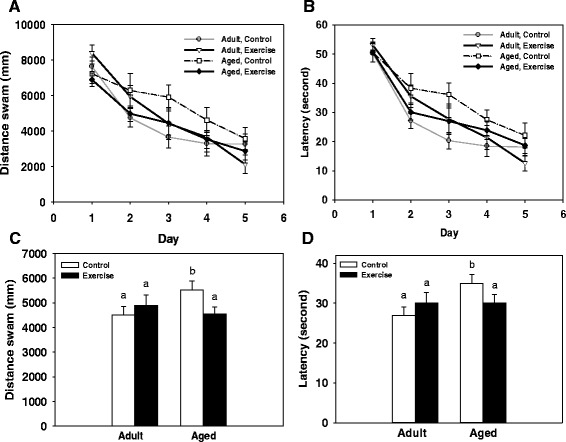
Spatial learning. Performance in the water maze was assessed by measuring distance swam (i.e., path length; mm) to locate the platform (**a**) and latency (seconds) to locate the platform (**b**). Lines represent the daily group average ± SEM. Overall, exercise reduced the distance swam (**c**) and latency (**d**) to locate the platform in the aged mice compared to aged controls. Data are collapsed across treatment condition. Bars represent the group average collapsed across the five test days ± SEM. Bars with different letters are significantly different from each other

**Fig. 3:**
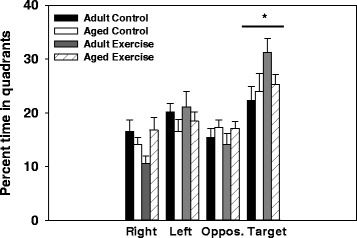
Probe trial. Analysis of the percent time spent in the four quadrants of the water maze during the probe trial showed that mice spent more time in the target quadrant when compared to all other quadrants. * indicates a significant difference in the percent time spent in the target quadrant compared to the right, left, and opposite (oppos.) maze quadrants. Bars represent group means ± SEM

A significant main effect of test day for distance swam (i.e., path length) to locate the hidden platform showed that all animals acquired the task as they reduced their distance swam across the five days of testing (F(4, 152) = 31.30, *p <* 0.0001, see Fig. 2a). In addition, a significant age x exercise interaction (F(1, 38) = 5.20, *p <* 0.05, see Fig. 2c) revealed that overall aged exercise mice swam a shorter distance to located the platform compared to aged control mice. Further, the aged control mice swam a longer distance than adult control mice (*p <* 0.05). The animal’s treatment condition (LPS or saline given one week prior to testing) had no effect on distance swam in either the adult or aged mice. Similarly a significant age x exercise condition interaction for latency to locate the platform (F(1, 38) = 6.67, *p <* 0.05, see Fig. 2b and 2d) showed that overall aged exercise mice had shorter latencies to locate the platform relative to aged controls. In addition, the aged control mice had longer latencies compared to the adult control mice (*p <* 0.05). A significant age × exercise × day interaction (F(4, 152) = 2.56, *p <* 0.05) for swim speed showed that adult exercise mice swam slower than adult control mice on day 3 of testing (data not shown). Analysis of the probe trial showed there was a main effect of quadrant (F(3, 114) = 20.02, *p <* 0.05, see Fig. 3), mice spent more time in the target quadrant (contained platform during training) than the other three quadrants (i.e., left, right, and opposite of target). However, an animal’s age or exercise condition had no effect on the number of platform crossings or the proportion of time spent in the target quadrant during the probe trial.

### New cell survival and new neurons

**Fig. 4:**
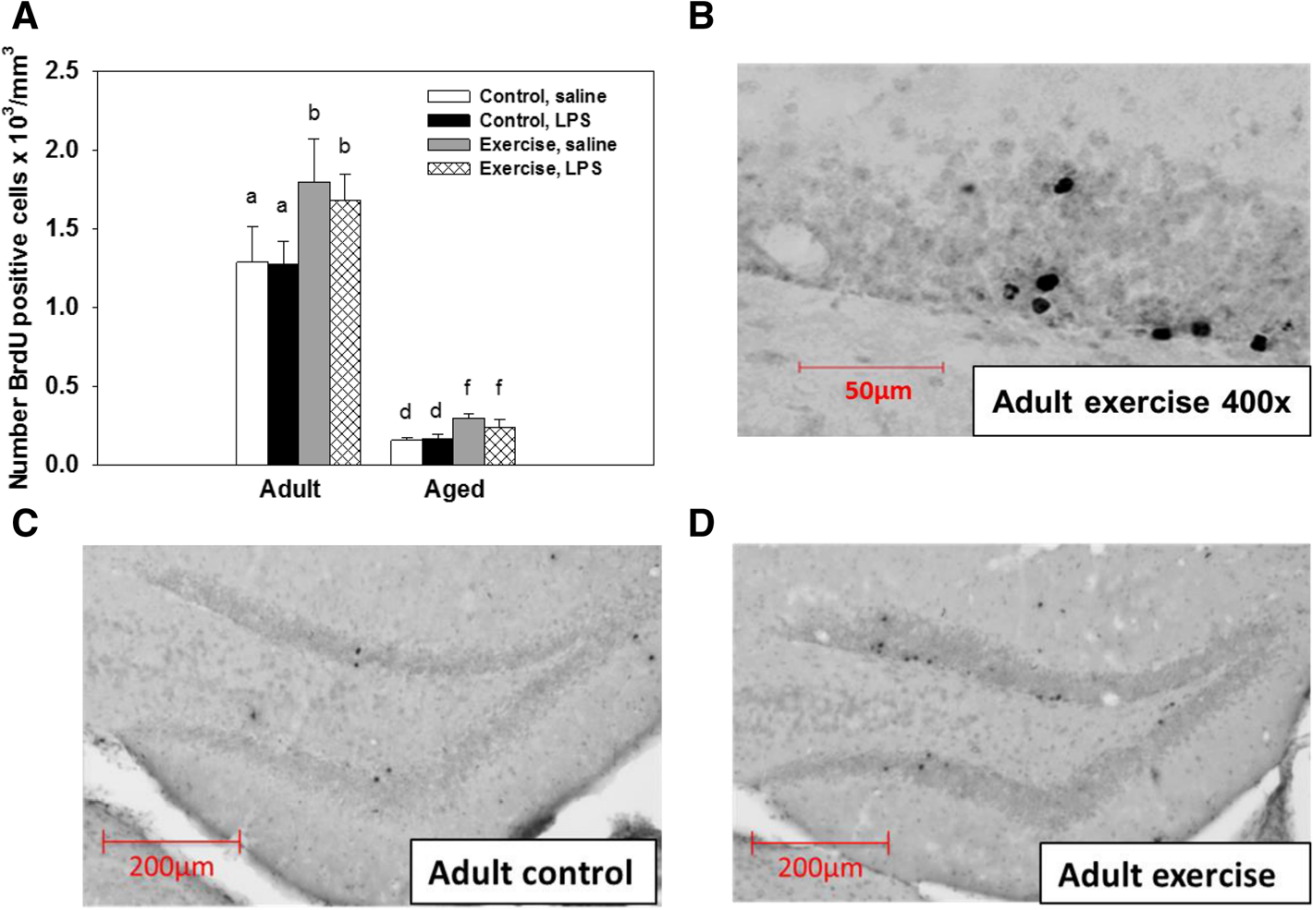
New cell survival. Number of BrdU positive cells in the granular cell layer of the hippocampus per cubic millimeter in adult and aged mice (**a**). For all conditions, aged mice showed reduced numbers of BrdU+ cells compared to adult mice. Wheel running increased the number of BrdU+ cells in both adult and aged mice. Representative images of BrdU positive cells in the granular layer of an adult exercise mouse at 400× magnification (**b**) adult control (**c**) and adult exercise (**d**) mice. Bars with different letters are significantly different from each other. Means ± SEM

**Fig. 5:**
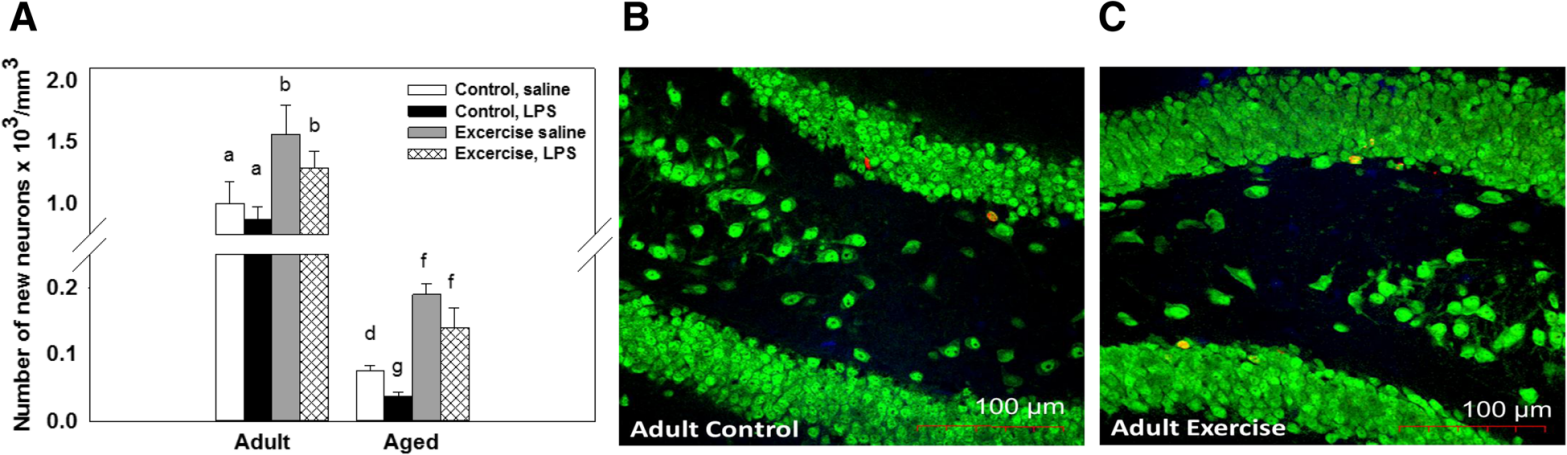
New neurons. Number of new neurons in the granular cell layer of the hippocampus of adult and aged mice (**a**). LPS decreased new neurons in the aged control mice, but had no effect in adults or aged exercise mice. Representative images of cells double labeled with antibodies against neuronal nuclei (mature neuron; green) and BrdU (new cell; red) from adult control (**b**) and adult exercise (**c**). Bars with different letters are significantly different from each other. Means ± SEM

There were significant main effects of age and exercise for the number of BrdU positive cells in the granular cell layer (F(1, 34) = 343.65, *p <* 0.001; F(1, 34) = 13.94, *p <* 0.001, respectively, see Fig. 4). Aged mice showed a reduced number of BrdU positive cells compared to adults. Exercise increased the number of BrdU positive cells in both adult and aged mice. LPS administration had no effect on the number of BrdU positive cells that survived in either the adult or aged mice. Comparison of the granular cell layer volume showed that there were no significant differences between any of the treatment groups. Assessment of the estimated number of BrdU positive cells co-expressing the mature neuron marker, neuronal nuclei, in the granular cell layer showed a significant main effect of age, exercise, and LPS administration (F(1, 34) = 559.99, *p <* 0.0001; (F(1, 34) = 51.80, *p <* 0.0001; F(1, 34) = 11.02, *p <* 0.01, see Fig. 5). Post hoc testing showed that LPS administration significantly decreased the number of new neurons in aged control mice relative to saline-treated aged controls (*p <* 0.01, see Fig. 5a). Whereas LPS did not reduce new neuron numbers in the adult mice or the aged exercise mice (see Fig. 5a). In addition there was a significant age × exercise interaction (F(1, 34) = 9.74, *p <* 0.01, see Figs. 5a) that showed exercise increased the number of new neurons in both adult and aged mice, but the adult mice still had a significantly greater number of new neurons when compared to the aged mice (*p <* 0.01).

### Proportion microglia positive for BDNF

**Fig. 6:**
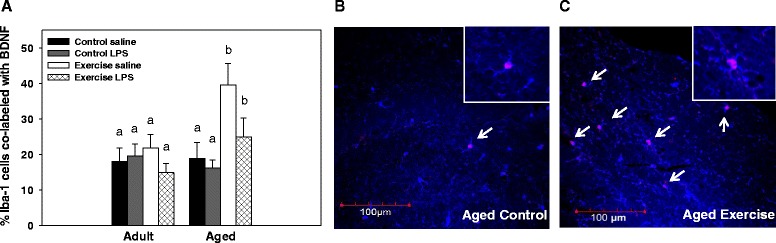
Proportion of BDNF positive microglial cells. Average percentage of Iba-1 positive cells that co-label with BDNF in the dentate gyrus of adult and aged mice (**a**). An age x exercise interaction showed that wheel running increased the proportion of BDNF and Iba-1 co-labeled cells only in the aged mice. Representative image of cells co-labeled with antibodies against Iba-1 (macrophage/microglia; blue) and BDNF (neurotrophic factor; red) from an aged control (**b**) and aged exercise (**c**) mouse. Arrows point to co-labeled cells. Bars with different letters are significantly different from each other. Means ± SEM

There was a significant age x exercise interaction for the proportion of microglia (i.e., Iba-1 positive cells) that co-labeled with BDNF (F(1, 34) = 6.94, *p <* 0.01, see Fig. 6a). Post hoc analysis showed that exercise increased the proportion of BDNF positive Iba-1cells in the aged, but not adult, mice. There was no difference in the proportion of co-labeled cells between the adult and aged control mice. There was a trend for LPS administration to reduce the proportion of BDNF positive Iba-1 cells, but this effect did not reach statistical significance (F(1, 34) = 3.93, *p* = 0.055).

### Correlations between proportion of BDNF positive microglia and new neuron numbers

**Fig. 7:**
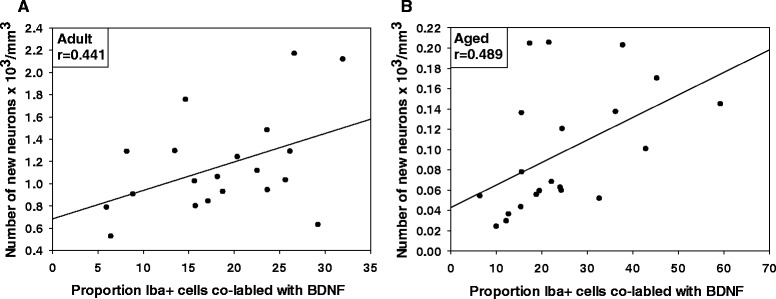
Correlations between BDNF positive microglia and neurogenesis. Correlations between the number of new neurons and the proportion of BDNF positive Iba-1 cells in adult (**a**) and aged (**b**) mice. Aged, but not adult, mice show a positive correlation between BDNF positive Iba-1 cells and the number of new neurons. Individual graphs list the Pearson’s correlation coefficient (i.e., r value)

To make the sampling regions comparable between the BDNF+ Iba-1+ cells and new neurons, only new neurons from the dorsal hippocampus (approximately −1.1 to −2.7 relative to bregma) were included in the correlation analysis. For the aged mice, there was a significant positive correlation between the number of new neurons and the proportion of BDNF and Iba-1 co-labeled cells (r =0.481, *p <* 0.05, see Fig. 7b). The correlation indicates that for the aged mice, higher proportions of BDNF positive Iba-1 cells were associated with an increased number of new neurons (see Fig. 7b). There was a trend for adult mice to show a correlation between new neuron numbers and the proportion of BDNF and Iba-1 co-labeled cells, but this effect did not reach statistical significance (r =0.441, *p =* 0.052 ns, see Fig. 7a).

## Discussion

Research has established that the form of microglia activation is a key factor in determining whether these cells support or disrupt hippocampal neurogenesis [5]. The present data extend these findings by showing that aged mice show greater reductions in new neuron production following an immune challenge relative to adults. Further, we report that wheel running prevented these LPS-induced reductions in neurogenesis in the aged mice. The beneficial effects of exercise may, in part, relate to its ability to induce a neuroprotective phenotype in microglia in the aged brain, as exercise increased the proportion of microglia that co-labeled with the neurotrophic factor BDNF. In the aged mice, the proportion of BDNF co-labeled microglia positively correlated with the number of surviving new neurons. Ultimately, our data demonstrate that normal aging results in a greater vulnerability to inflammation-induced reductions in neurogenesis and indicate that exercise may reduce this sensitivity.

Prior work has established that microglia in the aged brain are in a primed state [17, 29, 38]. As a result, aged subjects show increased basal levels of proinflammatory cytokines and prolonged neuroinflammation accompanied by greater deficits in cognitive function following an immune challenge [27, 39]. In addition, normal aging has been shown to enhance the sensitivity to inflammation-related deficits in neural plasticity. For instance, aged, but not adult, mice show dendritic atrophy following LPS administration, as aged mice show a significant reduction in the length and number of branches of apical dendrites of pyramidal neurons in the CA1 field [40]. Further, Chapman et al. [41] showed that prior infection with *E.coli* produced greater deficits in a specific form of late-phase long-term potentiation induced by theta bursts in hippocampal slices from aged rats when compared to adults. The present data complement these findings, as aged subjects showed increased sensitivity to LPS-induced reductions in hippocampal neurogenesis relative to adults. LPS decreased the number of new neurons in aged, but not adult, mice, indicating that immune activation can modify the fate of newly born cells. Prior work has shown that LPS reduces cell proliferation and cell survival [42, 43]. However, it is unlikely that LPS-induced reductions in proliferation or survival contributed to the observed decrease in new neurons in the aged subjects, as LPS had no effect on the number of surviving cells in the current study. Further, prior work has shown that LPS does not selectively disrupt survival of neuron precursors, as LPS-induced apoptosis occurs at a similar rate in doublecortin negative and doublecortin positive cells [6]. Therefore, the LPS-induced decrease in new neurons seen in the aged mice likely results from fewer cells differentiating into neurons rather than an immediate effect of LPS on survival or proliferation. Collectively, these data demonstrate that normal aging enhances susceptibility to impairments in hippocampal neurogenesis following an inflammatory insult.

Voluntary wheel running was able to attenuate the inflammation-induced impairments in neurogenesis in the aged mice. LPS administration decreased the number of new neurons in the aged mice; however, access to a running wheel for one month prior to LPS exposure prevented the LPS-induced reductions in new neurons. These protective effects of exercise in the aged are in agreement with prior work that found exercise prevented memory deficits following an *E.coli* challenge in aged rats [27]. Taken together, these data indicate that exercise may offer some protection against the increased sensitivity to immune activation in aged subjects.

The precise mechanism through which exercise exerts its beneficial effects within the brain has yet to be fully elucidated. However, prior research has identified multiple molecular changes that likely contribute to the exercise-induced enhancements in cognitive function and neural plasticity. For instance, exercise increases BDNF, a neurotrophic factor that enhances memory function and hippocampal neurogenesis [24, 22, 44–46]. However, the ability of exercise to increase BDNF may, in part, depend on IGF-1, as prior work has shown that inhibition of IGF-1 prevents the exercise-induced increase in BDNF [47]. Though excessive corticosterone levels can inhibit neurogenesis [48], the pro-neurogenic effects of BDNF may also depend on corticosterone. Prior work has shown that when diurnal corticosterone rhythms are disrupted, the ability of BDNF to increase neurogenesis is absent [49]. Further, Fuss et al. [45] have shown that running increases anxiety-like behavior, corticosterone levels, and hippocampal neurogenesis; potentially indicating that changes in corticosterone may contribute to the beneficial effects of exercise. Additional work indicates that the endogenous opioid system regulates hippocampal neurogenesis both basally and in response to exercise, potentially via its effects on the hypothalamic-pituitary adrenal axis [26]. Collectively, these data illustrate the diverse signaling pathways through which exercise may regulate hippocampal neurogenesis.

An additional possibility is that some of the beneficial effects of exercise are related to its ability to reduce inflammation by modulating microglia activation within the aged brain. Prior work has shown that exercise reduces hippocampal levels of IL-1β both basally and following an infection in aged rats [27]. However, others report that prior exercise training does not attenuate LPS-induced cytokine production within the whole brain of aged mice [50, 51]. Potentially, these differences may result from using whole brain versus hippocampal samples. As we previously reported wheel running decreased major histocompatibility class II expression on microglia isolated from the hippocampus, but had no effect on microglia isolated from the brain of aged females [29]. Further work is needed to determine whether exercise reduces inflammation in a region specific manner and if these changes contribute to increases in hippocampal neurogenesis.

Additionally, exercise may influence microglia by promoting the alternative microglia phenotype, as prior work has shown that wheel running increases hippocampal levels of the chemokine CX3CL1 that promotes a neuroprotective microglia phenotype [52]. Additionally, wheel running increases the proportion of IGF-1 positive microglia, a response that is enhanced in aged subjects [15]. The current data extend these findings by showing that exercise increases the proportion of microglia that co-labeled with BDNF in the aged brain. While several cell types secrete neurotrophic factors in response to exercise, prior work has established that BDNF and IGF-1 released by microglia are involved in cognitive function and stimulating regeneration [53, 14]. For instance, BDNF produced by activated microglia has been found to increase neurite growth *in vitro* [54]. Further, following an ischemic injury high proportions of IGF-1 positive microglia were associated with enhanced neurogenesis [12]. Selective inhibition of BDNF from microglia impaired memory in an auditory fear conditioning task as well as decreased learning-induced synapse formation, indicating that BDNF released from microglia can regulate select memory processes [14]. In the current study, aged mice showed a positive correlation between the proportion of BDNF co-labeled Iba-1 cells and the number of new neurons. Potentially, these exercise-induced changes in microglia activation may create a microenvironment that supports neuron production. In culture, research has shown that induction of the alternative neuroprotective form of microglia activation increases neurogenesis, as neural progenitor cells cultured with interleukin-4 stimulated microglia show increased neuronal differentiation as measured by doublecortin expression [7]. Beyond changes in microglia activation, prior work has shown that microglia density is negatively correlated with new cell proliferation and that wheel running reduces Iba-1 cell density in the dentate gyrus [55]. While altering microglial cell activity may not be the primary mechanism through which exercise increases neurogenesis, regulating these cells may be an important factor in enhancing neural plasticity within the aged brain. Though more work is needed to characterize the connection between exercise-induced changes in microglia activation and hippocampal neurogenesis, the current data indicate that exercise increases the proportion of microglia expressing a proneurogenic phenotype in the aged brain.

In contrast to the aged mice, there was no effect of exercise on the proportion of microglia that co-labeled with BDNF in the adult mice. Further, while exercise increased neurogenesis in the adult brain we found no evidence that microglia were involved, in agreement with prior work [31]. While microglia have previously been shown to modulate hippocampal neurogenesis in the adult brain, this effect appears to require a stimulus to activate microglia either towards the inflammatory or neuroprotective phenotype [7, 8]. Research by Olah et al. [31] demonstrated that wheel running does not induce any morphological or antigenic signs of microglia activation in the adult brain and concluded that exercise-induced increases in neurogenesis did not involve microglial cells. Microglia are known to support neurogenesis in the adult brain through maintaining the surrounding environment via removal of apoptotic new cells by phagocytosis [56]. However, the potential role of microglia in regulating cell differentiation in the adult brain is likely minimal in the absence of an injury or activating stimulus whereas microglia in the aged brain appear to participate in regulating basal levels of neurogenesis [19]. The age-associated priming of microglia may increase responsiveness to exercise-induced changes in the environment, whereas microglia in the adult brain may need to undergo activation before they react. The current data support this contention as exercise increased the proportion of microglia that co-labeled with BDNF in the aged brain, but had no effect in the adults. Potentially, activated microglia may be more plastic than non-activated cells.

In the present study LPS administration had no effect on the survival of new cells or the number of new neurons in the hippocampus of adult mice. These data are in agreement with the findings of Bastos et al. [42] who report that LPS did not alter the number of new cells that co-labeled with the neuron markers doublecortin or neuronal nuclei. However, prior reports have shown that immune activation can decrease neuronal differentiation. For instance, Monje et al. [6] found that systemic LPS administration in adult rats reduced the proportion of doublecortin positive new cells, indicating that LPS reduces neuronal differentiation. Similarly, 4 weeks of intracortical LPS administration reduced new neuron production [8]. The duration and dose of LPS likely contributes to the variation in findings. In the present study, we administered a single systemic LPS injection at a dose of 250 μg/kg whereas Monje et al. [6] who reported reductions in neuronal differentiation gave an LPS dose that was four times higher (i.e., 1 mg/kg). Similar dose dependent effects of LPS have also been reported for cell proliferation and survival, as LPS at 1 mg/kg, but not lower doses, reduced cell proliferation and the number of surviving cells in adults [42, 43, 56]. Collectively the data indicate that adults require high doses of LPS to induce alterations in neurogenesis.

Through the course of the experiment wheel running behavior was altered by the experimental manipulations. As expected, LPS administration transiently reduced distance ran in both adult and aged mice when compared to saline-treated mice. However, after the effects of LPS dissipated, all groups maintained a lower average running distance during the last month of the experiment. Prior reports have shown that if left undisturbed mice maintain a similar level of running for up to 50 days, however, if subjected to behavioral testing wheel running decreases [2, 35]. This reduction in wheel running likely reflects a combination of the physical requirements (i.e., swimming) and the stressful nature of the water maze task. Prior research has shown that water maze testing increases corticosterone levels [57]. While the ability of exercise to modulate the response to stress has been well studied [58, 59], how stress impacts exercise behavior has not been elucidated. Though a direct assessment of how stress may modulate exercise is needed, the current data in conjunction with prior reports indicates that stressful experiences may reduce wheel running [2, 34].

In summary, normal aging enhances sensitivity to LPS-induced reductions in hippocampal neurogenesis, as aged mice show fewer new neurons at a dose of LPS that did not alter neurogenesis in adult mice. Voluntary wheel running can alleviate the age-related reductions in new neurons in response to LPS. The ability of exercise to prevent the LPS-induced neurogenesis deficits in the aged mice may in part result from alterations in microglia activation. Exercise increased the proportion of BDNF co-labeled microglia. Ultimately, these data indicate that exercise increases microglia expression of a proneurogenic phenotype and attenuates the vulnerability to inflammation-induced deficits in hippocampal neurogenesis in the aged brain.

## Abbreviations

BDNF: Brain derived neurotrophic factor
BrdU: Bromodeoxyuridine
Iba-1: Ionized calcium-binding adapter molecule 1
IGF-1: Insulin-like growth factor-1
IL-1β: Interleukin-1β
LPS: Lipopolysaccharide
SEM: Standard error of the mean
TBS: Tissue buffering solution
